# Genetic rhabdomyolysis within the spectrum of the Spinocerebellar Ataxia type 2 responsive to pregabalin

**DOI:** 10.1186/s40673-021-00131-7

**Published:** 2021-03-05

**Authors:** Fabian Rossi, Joe Ma, Nina Tsakadze, Lourdes Benes-Lima, Julio Araque Gonzalez, Michael Hoffmann

**Affiliations:** 1 Department of Neurology, Orlando VA Medical Center, 32827 Orlando, FL USA; 2 Department of Neurology, University of Central Florida Medical School, 32827 Orlando, FL USA; 3 Department of Pathology, Director Neuromuscular Department, Florida Hospital, 32803 Orlando, FL USA; 4 Department of Neuroradiology, Orlando VA Medical Center, 32827 Orlando, FL USA

**Keywords:** Spinocerebellar ataxia, Rhabdomyolysis, Cramps, Pregabalin

## Abstract

**Background:**

Spinocerebellar Ataxia type 2 is a slowly progressive adult onset ataxia with a broad clinical presentation.

**Case presentation:**

We describe a man with Spinocerebellar Ataxia type 2 with chronic, severe, and recurrent rhabdomyolysis, as part of the cerebellar ataxia genetic spectrum. Initially rhabdomyolysis was refractory to multiple medications, but entirely resolved and remained in chronic remission with pregabalin.

**Conclusions:**

This is the first report of Spinocerebellar Ataxia type 2 associated with chronic, severe, recurrent rhabdomyolysis as part of its genetic phenotype responsive to pregabalin.

## Background

Spinocerebellar Ataxia type 2 (SCA2) is a neuro-degenerative ataxia with a heterogeneous clinical presentation, including both cerebellar (ataxia, dysarthria, slow horizontal saccades and dysmetria) and non-cerebellar features (peripheral neuropathy; extrapyramidal symptoms, sleep and cognitive disorders). Neuromuscular features such as cramps, fasciculations, and amyotrophy were often described within the spectrum of SCA2, but rhabdomyolysis has not been reported previously.

We report a case of SCA2 with chronic, severe, recurrent rhabdomyolysis as part of its genetic phenotype responsive to pregabalin.

## Case presentation

**Fig. 1:**
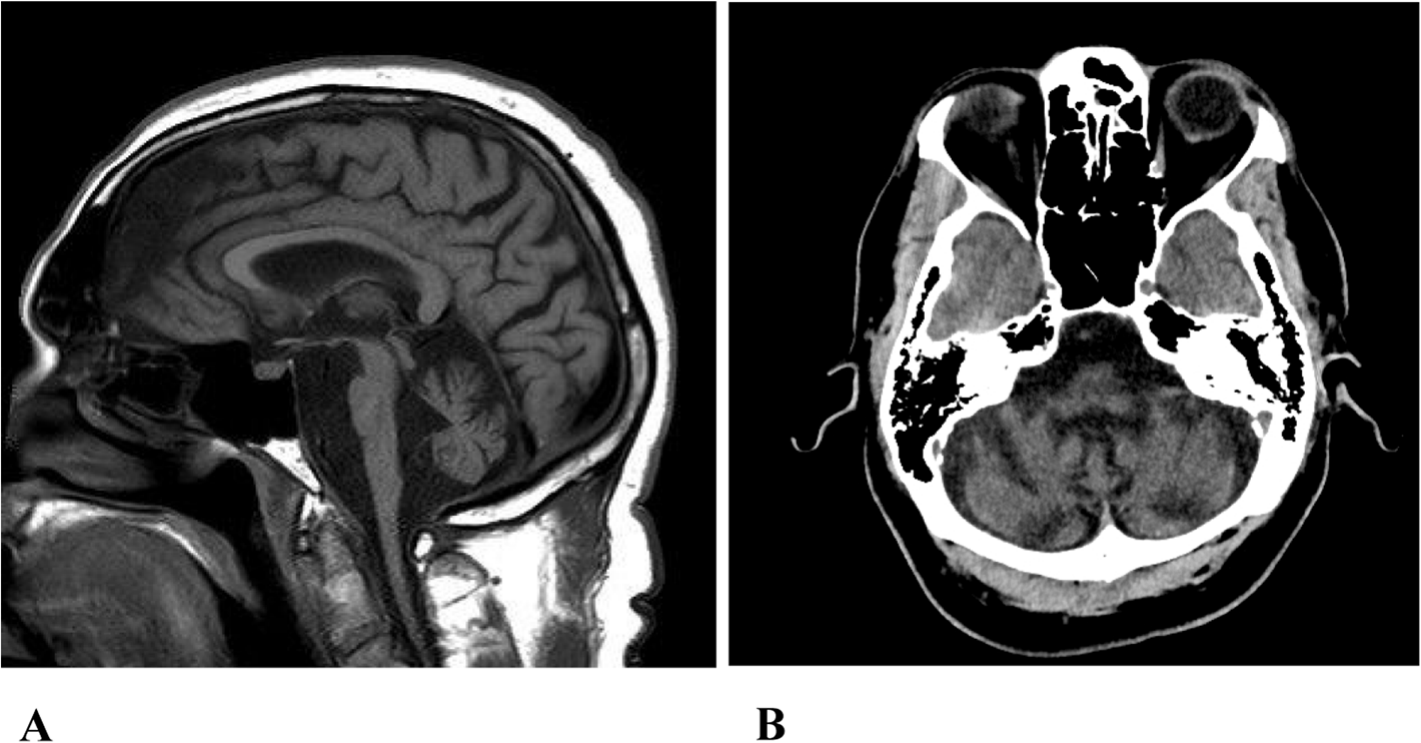
**a **Sagittal T1-weighted MRI brain demonstrating atrophy of the brainstem and cerebellum with increment in cerebellar folia. **b. **Axial CT head without contrast  demonstrating atrophy of the cerebellar hemispheres with prominent sulci.

**Fig. 2:**
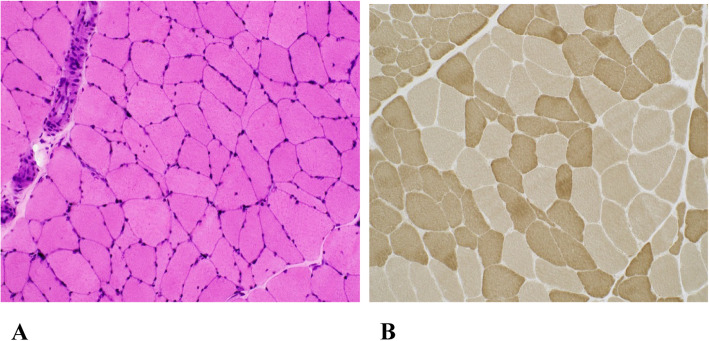
Cryosections of muscle biopsy showed scattered atrophic, polygonal and angulated myofibers without inflammation, myofiber necrosis or pathologic inclusions (H&E stain, 200x) (**a**) and myofiber type grouping characteristic of chronic neurogenic atrophy (myosin ATPase activity stain at pH 9.4, 200x) (**b**).

63-year-old black man, working in construction, presented with three-decade- history of intermittent muscle cramps. Cramps initially were only triggered by strenuous physical activity under heat and resolved after few minutes of rest, especially with cool temperature. Cramps affected his calves, quadriceps, and arm muscles. Symptoms started to deteriorate in the last two decades, when cramps started occurring spontaneously, more frequently and associated with elevation in creatinine kinase between 4000 and 12,000 IU/L, requiring hospitalization 3–4 times per month. At one time his creatinine kinase (CK) increased up to 26,171 IU/L with severe muscle cramps and pain. He also developed fasciculations in his limbs, abdomen, and back muscles; and atrophy of the right leg muscles below the knee. At the age of 40, when his cramps worsened, he developed progressive ataxia, dysmetria, dysarthria, dysphagia, and problems with reading. At the age of 58, he became wheelchair-bound and developed cognitive problems and bradyphrenia. He suffered from atrial fibrillation, deep venous thrombosis, diabetes, hypertension, and chronic untreated asymptomatic hepatitis C with normal liver function caused by IV drug abuse in his teen years. His medications were insulin, dabigatran, diltiazem, lamotrigine, omeprazole, sotalol, riboflavin, and most recently riluzole for ataxia. His grandmother, mother, and sister suffered from ataxia and painful incapacitating cramps that required multiple hospitalizations. General examination was unremarkable. Neurological examination showed slow horizontal and vertical saccades, dysarthria, bilateral dysmetria, dysdiadochokinesia, severe ataxia, right leg atrophy and generalized areflexia. He displayed a cerebellar deafferentation syndrome with executive dysfunction. Brain MRI showed pan-cerebellar atrophy (Fig. 1a and b). Laboratory tests including metabolic panel, vitamin levels, endocrine panel and toxins were negative. Mayo paraneoplastic panel, HIV, ANA, Sjogren panel, ceruloplasmin, Whipple’s polymerase chain reaction (PCR), myositis panel, angiotensin converting enzyme (ACE), anti-cyclic citrullinated peptide (anti-CCP), antiphospholipid and anticardiolipin antibodies, C-reactive protein (CRP), sed rate (ESR), and urine drug screen were all negative. Nerve conduction studies and electromyography (EMG) showed sensory-motor axonal loss polyneuropathy and non-specific myopathic features. The spinocerebellar ataxia type 1 (SCA1) and spinocerebellar ataxia type 3 (SCA3) DNA test, Mayo Clinic paraneoplastic panel, and glutamic acid decarboxylase (GAD 65) were all negative. The SCA2 DNA test showed 39 CAG repeats in the ataxin-2 gene (ATXN2 gene). A right quadriceps muscle biopsy at age 54 showed chronic neurogenic atrophy and no associated inflammation (Fig. 2a and b). Muscle biopsy was otherwise negative for inflammatory myopathies, vasculitis, muscular dystrophies, glycogenosis, lipid storage disease, and mitochondrial myopathies.

He failed the following preventive medications for painful muscle cramps and rhabdomyolysis: quinine, gabapentin, clonazepam, diazepam, creatinine, and vitamin B. Pregabalin at 150 mg twice daily remitted all his cramps and rhabdomyolysis attacks for more than four years. His CK values between attacks remained between 400s and low 1,000s,

## Discussion

SCA2 is a rare autosomal dominant cerebellar ataxia that affects 5–7 per 100,000 inhabitants with a worldwide distribution. In some region of the world (northeastern Cuba) the prevalence is ten times higher. SCA2 is the second most common spinocerebellar ataxia after Spinocerebellar Ataxia type 3 [1]. SCA2 is caused by CAG trinucleotide repeat expansions in the ATXN2 on chromosome 12q24. The abnormally translated ataxin-2 proteins have expanded polyglutamine (polyQ) tracts with an increased tendency to aggregate and to interact abnormally with other cellular proteins, leading to abnormal RNA processing, a general suppression of gene transcription, and cell death [2]. Cerebellar ataxia with onset in adulthood occurs when there are 33 or more CAG repeats [3]. Two hundred or more CAG repeats are implicated in infantile presentation with developmental delay, infantile hypotonia, and retinitis pigmentosa [4]. It presents with progressive cerebellar truncal ataxia, dysmetria, and dysarthria. Slow horizontal saccades (reported in 90 % cases) and peripheral polyneuropathy with hyporeflexia or areflexia facilitates its diagnosis and help to differentiate it from other cerebellar ataxias [1–3]. A form of SCA2 presenting as dopa-responsive parkinsonism or with supranuclear palsy-like phenotype was described in China [5]. Pathological studies reveal atrophy of the cerebellum, brainstem, frontal lobe, and white matter [2, 3]. SCA2 is commonly associated with neuromuscular involvement. A sensory and motor polyneuropathy is present in 90 % of patients. Fasciculation and amyotrophy are also common, though amyotrophy is more common in advanced disease [1]. An increased risk for motor neuron disease was reported in intermediate alleles of ATXN2 gene [2]. Painful muscle cramps were seen in 80 % of patients of a large series of SCA2 from Cuba [6]. Cramps were thought to result from distal motor neurons hyper-excitability, caused by collateral sprouting from the axonal damage [1]. However, rhabdomyolysis has never been reported previously as part of the SCA2 spectrum. It was not clear if his repetitive severe muscle cramps caused the rhabdomyolysis or on the contrary if his cramps were a clinical manifestation of his repetitive rhabdomyolysis. We suspect that his cramps resulted from his repetitive attacks of rhabdomyolysis, since his cramps resolved as the hyper-CK-emia normalized. Hepatitis C was also implicated in rhabdomyolysis, but always in combination with other triggering factors, such as cocaine or heroin abuse, and concurrent use of anti-hepatitis C viral therapy [7–9]. Hepatitis C was associated with perivascular infiltrative lymphocytes and muscle fibers invasion that resulted in myositis [10–12]. However in our case, the role of hepatitis C in rhabdomyolysis remains unclear since muscle biopsy showed no evidence for myositis, there was only a very distant history of illicit drug abuse during his teens, but not for decades [based on the history and repetitive urine analysis tests over the years] and he never received therapy for hepatitis C. It is conceivable that hepatitis C in combination with genetic disorder with predisposition to rhabdomyolysis, such as SCA2, was the cause for these repetitive attacks of rhabdomyolysis. No other etiology or trigger factor was identified.

In this case, pregabalin caused a total remission of the cramps, fasciculations, and rhabdomyolysis attacks, with normalization of baseline CK for his race and body habitus [values oscillated between 400s and 1000s] [13], however cerebellar dysfunction persisted. The selection of pregabalin was based in its membrane stabilizing effect and prior sporadic cases reports where pregabalin was effective to treat fasciculation and cramps syndrome and unreported cases seen by authors [14, 15].

In summary, this is the first report of rhabdomyolysis, as part of the SCA2 spectrum. This case suggests, that rhabdomyolysis should be suspected in each patient with SCA2 who complains of persistent myalgias and painful muscle cramps. The role of the hepatitis C in this case remains unknown. Pregabalin should be considered, as an early treatment for myalgias, muscle cramps, and rhabdomyolysis.

## Data Availability

non-applicable.
